# Placental Cortisol Dysregulation in Mothers with Experiences of Childhood Adversity: Potential Mechanisms and Clinical Implications

**DOI:** 10.3390/jcm13072020

**Published:** 2024-03-30

**Authors:** Joshua George, Maria Muzik, Courtney Townsel

**Affiliations:** 1 Department of Obstetrics and Gynecology, University of Michigan, Ann Arbor, MI 48109, USA; 2 Department of Psychiatry, University of Michigan, Ann Arbor, MI 48109, USA; muzik@med.umich.edu; 3 Department of Obstetrics, Gynecology and Reproductive Health Sciences, University of Maryland, Baltimore, MD 20742, USA; ctownsel@som.umaryland.edu

**Keywords:** adverse childhood experiences, cortisol, HPA axis, childhood trauma, maternal health

## Abstract

Adverse childhood experiences (ACEs) are extremely prevalent in the United States population. Although ACEs occurs in childhood, exposure to them has been associated with adverse future pregnancy outcomes and an increased risk of poorer social determinants of health, which further drive the risk of negative pregnancy outcomes. In addition, maternal ACE exposure has been linked to poor infant and child outcomes, highlighting the intergenerational transmission of risk from mother to child. While alterations along the Maternal–Placental–Fetal Hypothalamic–pituitary–adrenal (HPA) axis is hypothesized to be involved, the exact biological pathway underlying this intergenerational passage of risk is mostly unknown. This present work will highlight what is known about pregnancy-related stress hormone physiology, discuss the potential mechanisms of action of ACEs on cortisol regulation, and suggest opportunities for further clinical and translational studies.

## 1. Introduction: The Maternal–Placental–Fetal Hypothalamic–Pituitary–Adrenal (HPA) Axis Regulating Fetal Stress

The hypothalamic–pituitary–adrenal (HPA) axis is the main stress response system in the human body. It regulates the body’s adaptation to stress and maintains day-to-day homeostasis. Proper functionality of the HPA system is crucial to physical and mental health. During pregnancy, the placental corticotropin-releasing hormone (CRH) acts as a stimulator of the maternal HPA axis, leading to the production of cortisol, a stress hormone. In normal physiology, the levels of placental CRH increase throughout pregnancy and reach their peak near term. Fetal exposure to high levels of circulating glucocorticoids, which may result from the upregulation of placental–fetal stress physiology, has been shown to have negative effects on infant HPA regulation and subsequent socioemotional development [1].

Fetal cortisol exposure consists of its intrinsic production by the fetal adrenal gland and exposure to maternally derived cortisol. Physiologic cortisol exposure is vital to the development of the fetus’s lungs, thyroid, gastrointestinal tract, and glucose regulation pathways. The fetus is protected from excess cortisol exposure through the regulation of the placental enzyme 11-Beta Hydroxysteroid Dehydrogenase-2 (11β-HSD2), but this regulatory pathway may be impacted by maternal stress or enzyme-inhibiting factors [2].

The primary mechanism of protecting the fetus from excessive cortisol exposure in utero is an increase in the expression of placental 11β-HSD2, an enzyme expressed by syncytiotrophoblast cells that converts cortisol into its inactive form, cortisone, via oxidation. This enzyme is capable of metabolizing approximately 80–90% of maternally derived cortisol that crosses the placenta. In normal physiology, its expression is decreased in the third trimester. This allows for increased cortisol transfer across the placenta to facilitate physiologic fetal organ development and fetal glucose demands. Synthetic glucocorticoids given for medical indications or excess maternal cortisol due to stress may overwhelm this natural protective mechanism, especially during the physiological nadir of placental 11β-HSD2 in the third trimester [2].

## 2. The Role of Adverse Childhood Experiences on Placental–Fetal Stress Hormone Cortisol Regulation

**Figure 1:**
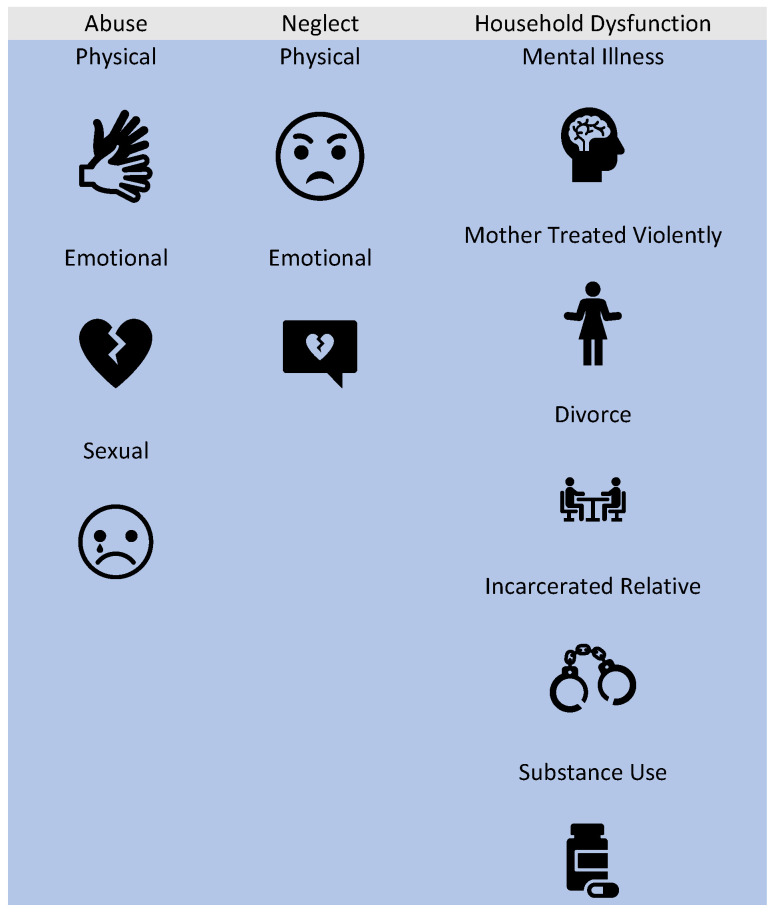
Categories and types of adverse childhood experiences.

Adverse childhood experiences (ACEs), which encompass preventable and potentially traumatic events occurring before the age of 18, are alarmingly prevalent [3]. According to the 2011–2020 Behavioral Risk Factor Surveillance System monitored by the Centers for Disease Control, approximately 63.9% of U.S. adults reported having experienced at least one ACE [4]. ACEs can be classified into three broad categories, including household dysfunction, abuse, and neglect (Figure 1). Additionally, experiencing four or more ACEs was more common among females (19.2%) than males (15.2%) [4]. Individuals who have experienced ACEs are at a higher risk of negative pregnancy outcomes, such as hypertensive disorders of pregnancy, a low birthweight, and preterm births [5]. Furthermore, these individuals face increased adverse social determinants of health, which further compound the likelihood of negative pregnancy outcomes [5]. While the original ACE study also determined that specific categories of ACEs have greater impact on the health effects, this same categorical effect is yet to be determined for pregnancy outcomes. It also remains unknown whether ACEs have a dose-dependent relationship with negative pregnancy outcomes. It is important to note that the impact of ACEs extends beyond the immediate health consequences for the affected individual. Maternal ACE exposure, for instance, has been linked to poor infant and childhood outcomes, underscoring the potential for the intergenerational transmission of risk from mother to child [6]. This cycle of risk highlights the urgent need to understand the molecular underpinnings of intergenerational transmission and its influences on future maternal and child health.

In addition to direct effects on pregnancy outcomes, ACEs have also been associated with an increased likelihood of engaging in risky health behaviors during pregnancy. Pregnant people who have experienced ACEs are more likely to smoke tobacco, use illicit substances, and have inadequate prenatal care [7]. These behaviors further contribute to negative outcomes for both the birthing individual and the developing fetus [8]. In this present work, we explore what is known about the physiology of ACEs in the maternal–infant dyad and will focus on how dysregulation of the HPA axis pathway due to ACEs might lead to adverse pregnancy outcomes. It is worth noting that there are multiple other hypothesized mechanisms of intergenerational transmission of stress that have been described as alternatives to or mediators or moderators of the HPA axis mechanism. Chronic or prenatal stress has been described in the activation of the sympatho-adrenomedullary system, maternal immunosuppression and proclivity to infection, alternation of the intestinal microbiota, and disruption of healthy eating, sleep, and the postnatal environment [9]. While this piece will primarily focus on the impact of ACEs on HPA axis regulation, it is important to consider that these other mechanisms may contribute some effects that may be relevant to understanding how ACEs impact pregnancy and influence future health.

**Figure 2:**
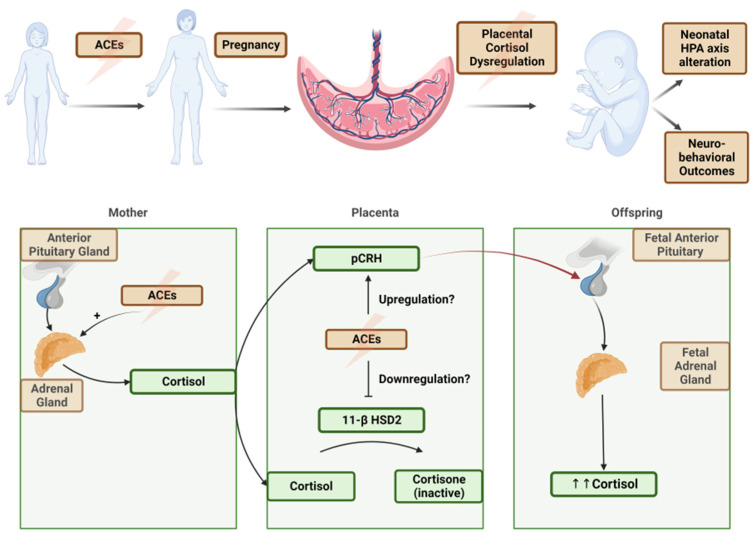
(**above**) A theoretical model of transgenerational impact of ACEs. (**below**) Hypothesized molecular pathways of ACE upregulation of maternal stress hormones, placental cortisol pathway dysregulation, and a net increase in fetal cortisol exposure.

ACEs are associated with the upregulation of placental–fetal stress physiology through placental corticotropin-releasing hormone [10] (Figure 2). This suggests a proposed pathway from maternal trauma exposure and a dysregulated HPA axis during pregnancy to “toxic” fetal glucocorticoid exposure, resulting in infant HPA dysregulation and impaired development. However, the exact biological pathway underlying this intergenerational passage of risk is unknown. Previous research has confirmed that maternal ACEs are associated with higher levels of tonic and diurnal cortisol parameters and a blunted phasic response to acute stressors [11].

Childhood trauma exposure has been linked to long-lasting disruptions in HPA functionality in trauma-exposed individuals, leading to an increased risk of adverse health outcomes, ranging from mental health conditions and addiction to cardiometabolic diseases [12]. As these diagnoses are independently associated with adverse pregnancy outcomes such as preterm births and preeclampsia, this suggests that childhood trauma exposure may have some downstream effect on future pregnancy outcomes. Furthermore, adaptations in the HPA axis that alter cortisol may affect vital fetal programming functions, including fetal organ development and neurodevelopment and the timing of parturition [13].

The existing research has focused on the associations between maternal antenatal stress, the HPA axis, and fetal programming. As described above, alterations in the maternal and fetal HPA axis have been connected to altered neonatal cortisol and neurodevelopmental outcomes, but our understanding of the molecular targets is less thorough. Placental 11β-HSD2 has been a primary target of study and has been implicated as a target for downregulation in pregnant people with increased psychosocial stress, anxiety, and depression [14]. Other molecular pathways, including the sympathetic nervous system and the inflammatory response system, have been investigated, but those data are not as consistent [15]. Given these findings, it is suggested that the impairment of placental 11β-HSD2 regulation is potentially greater for those who have experienced ACEs, resulting in increased fetal cortisol levels at an earlier gestational age. While acute stressors may be short-lived or mitigated, the effect of ACEs may be entrenched well before pregnancy occurs. Further study to validate this mechanism and its downstream clinical ramifications is needed.

## 3. Connecting the Dots between ACEs and Placental Cortisol Regulation via 11-Beta Hydroxysteroid Dehydrogenase (11β-HSD2)

Previous studies have established that placental 11β-HSD2 is sensitive to maternal prenatal mood disorders. Seth et al. and O’Donnell et al. demonstrated that the expression of placental 11β-HSD2 is downregulated in the presence of maternal mood disorders such as depression and anxiety [16]. The effect of trait or long-standing anxiety was noted to be independent of confounders such as age, parity, or substance use [17]. While these studies began to link placental molecular changes to maternal mood disorders, they did not investigate their impact on the cortisol levels in the umbilical cord tissue or umbilical cord blood to validate the downstream effects of enzyme regulation. Umbilical cord blood cortisol is an established proxy for fetal exposure to stress hormones and is linked in the literature to adverse infant outcomes [18,19]. In addition, the impact of long-standing stress exposure was not accounted for.

The impact of ACEs on placental 11β-HSD2 remains unclear. The influence of ACEs may occur much earlier as a form of chronic stress exposure from childhood. Animal models have shown a link between chronic stress exposure and an impaired ability to upregulate placental 11β-HSD2 in response to acute stress, diminishing the innate protective mechanism against fetal steroid overexposure [20]. While human studies to validate this same response are needed, this maladaptation may have exponential effects when considering the increased likelihood of ACE exposure, co-existing mood disorders, and an impaired ability to regulate placental 11β-HSD2 in response to acute stress.

The mechanism linking the distant exposure of ACEs to adverse outcomes in offspring, or a transgenerational effect, is also yet to be fully elucidated. One hypothesized pathway is the epigenetic transcriptional repression of target genes via DNA methylation. In a cohort of offspring born to mothers with post-traumatic stress disorder (PTSD) from war-related trauma, preliminary data looking at the DNA methylation in candidate genes, including cortisol pathway genes, showed higher cortisol levels and differences in DNA methylation, although they did not reach statistical significance [21]. Overall, this suggests that the effects of trauma may be passed through generations via the epigenome. ACE exposure may act according to a similar mechanism. That is, 11β-HSD2 methylation resulting in transcriptional gene repression may result in decreased amounts of protective placental 11β-HSD2 enzyme, causing the fetus to be exposed to higher cortisol levels throughout pregnancy [22]. These changes may be subsequently transferred to future generations. If this epigenetic mechanism is proven, it is also essential to understand whether these changes are static or dynamic from the time of birth onwards or whether they act as the first strike in a “two-hit” model, creating vulnerability to future life stress. Validation of these molecular pathways is essential for understanding the extent of the impact of ACEs on maternal and fetal health, as well as possible transgenerational effects. In order to advance towards clinical utilization, further translational studies to substantiate the linkage between fetal cortisol exposure and placental 11β-HSD2, followed by a greater understanding of epigenetic influences, is needed.

## 4. Clinical Implications

Unlike other mental health conditions, such as depression, which is routinely screened for in obstetric care, ACE screening during pregnancy is not consistently performed. As a result, prior trauma often goes unrecognized, missing an opportunity for intervention. Additionally, the negative health and pregnancy outcomes associated with ACEs are only beginning to be explored.

Gaining an accurate physiologic understanding of how ACEs can adversely affect pregnancy and the health of offspring would provide an evidence-based rationale for implementing ACE screening as part of routine obstetric care. It would also advance our scientific understanding of the biological mechanisms behind the transmission of historical trauma from mother to child and promote the health of future generations by enabling risk stratification of mothers and neonates who may benefit from early interventions. Furthermore, the success of future targeted interventions for mothers with ACEs depends on first understanding these physiologic mechanisms. One intervention could include determining what types of resources (i.e., trauma-informed counseling, coping therapies, psychopharmacotherapy) would be of greatest benefit in understanding how this unique form of stress impacts physiology. Another future intervention could be a novel therapeutic that acts on placental 11β-HSD2 to modulate its function to ensure the protection of the fetus from excessive cortisol exposure. Understanding the complex relationship between ACEs and pregnancy outcomes is crucial for healthcare providers and policymakers as well. When considering primary prevention, identifying the intergenerational consequences od ACEs would lead credence to larger public health efforts like criminal justice reform, comprehensive substance use policy, and partner violence prevention, all of which are common forms of household dysfunction that lead to ACEs.

## 5. Conclusions

ACEs represent an increasingly prevalent and catastrophic underpinning for adverse health and pregnancy outcomes. The existing literature has limited data on the molecular mechanisms of ACEs’ impact and transgenerational transmission. We have outlined a potential mechanism connecting maternal ACEs, placental 11β-HSD2, and fetal stress regulation as the basis for the intergenerational origins of HPA dysregulation. Further work is needed to elucidate these pathways and inform personalized clinical interventions for vulnerable people.

## Data Availability

No new data were created or analyzed in this study. Data sharing is not applicable to this article.
